# Propolis vs. Chlorhexidine Gluconate and Benzydamine Hydrochloride on Sequelae After Third Molar Surgery: A Randomized Controlled Trial

**DOI:** 10.3390/ph19101615

**Published:** 2026-10-12

**Authors:** Zeynep Bayramoğlu, Zekai Halıcı, Emrah Özakar, Rukiye Sevinç Özakar

**Affiliations:** 1 Department of Oral and Maxillofacial Surgery, Faculty of Dentistry, Ataturk University, Erzurum 25240, Turkey; 2 Department of Pharmacology, Faculty of Medicine, Ataturk University, Erzurum 25240, Turkey; hzekai@atauni.edu.tr; 3 Department of Pharmaceutical Technology, Faculty of Pharmacy, Ataturk University, Erzurum 25240, Turkeyrukiyeso@atauni.edu.tr (R.S.Ö.)

**Keywords:** propolis, chlorhexidine, impacted third molar, wound healing, edema control

## Abstract

**Background.** Impacted lower third molar surgery is characterized by postoperative sequelae such as pain, edema, and trismus. While chlorhexidine (CHX) is the gold-standard antiseptic, its side effects prompt the search for natural alternatives. The aim of this study is to examine the effects of propolis gargle on wound healing following third molar surgery and to compare it with chlorhexidine gluconate and benzydamine hydrochloride (CHX+BENZ). **Methods.** In this prospective, randomized, double-blind, active-controlled trial, 52 patients were assigned to 3% Propolis (*n* = 24) or 0.12% CHX+BENZ (*n* = 28) groups. Mouthwashes were applied twice daily from postoperative days 2 to 7, and pain (VAS), edema, and trismus were evaluated preoperatively and on days 2 and 7. Statistical significance was set at *p* < 0.05. **Results.** No significant differences were found in pain and trismus (*p* > 0.05). However, the Propolis group exhibited significantly lower edema on day 2 compared to the CHX+BENZ group (*p* = 0.004), and complication rates were also notably lower in the Propolis group. **Conclusions.** The novel, non-alcoholic 3% propolis mouthwash demonstrated no statistically significant differences in short-term pain and trismus outcomes compared to CHX+BENZ, alongside a statistically smaller day-2 facial edema measurement. While these findings support its potential as a supportive oral rinse following third molar surgery, adequately powered prospective trials are warranted to validate these preliminary observations before routine clinical adoption.

## 1. Introduction

Surgical removal of impacted mandibular third molars is a routinely performed outpatient procedure requiring mucosal incision, flap elevation, and osteotomy. This surgical trauma triggers localized acute inflammation, clinically manifested as pain, facial edema, and trismus, which significantly impair early postoperative quality of life [1,2,3]. Consequently, identifying optimized pharmacological or therapeutic interventions to mitigate these complications remains a primary research priority [1,2,3,4,5].

To minimize surgical site microbial colonization and postoperative plaque accumulation, chemical mouthwashes are routinely prescribed alongside mechanical oral hygiene. Chlorhexidine gluconate (CHX)—often combined with benzydamine hydrochloride—remains the gold-standard oral antiseptic due to its broad-spectrum antimicrobial efficacy and high substantivity. However, prolonged CHX use is constrained by adverse effects, such as taste alterations, mucosal desquamation, and extrinsic tooth staining, while recent in vitro reports of cytotoxicity toward gingival fibroblasts and osteoblasts have driven the search for safer, natural bioactive alternatives [6,7].

Propolis, a resinous substance produced by honeybees (*Apis mellifera*), possesses potent antibacterial, antifungal, antiviral, and regenerative properties, making it widely used in dentistry and apitherapy. Caffeic acid phenethyl ester (CAPE) is its primary active constituent and, due to its lipophilicity, readily crosses cell membranes, downregulating the arachidonic acid cascade via selective cyclooxygenase (COX) and lipoxygenase (LOX) inhibition. Consequently, CAPE suppresses pro-inflammatory prostaglandins and leukotrienes responsible for pain and vascular permeability. Concurrently, it upregulates anti-inflammatory cytokines (IL-4, IL-10) and restricts neutrophil and monocyte infiltration into damaged tissues [8,9,10].

While the antimicrobial efficacy of propolis against oral pathogens is well documented, its comparative anti-inflammatory outcomes relative to standard chemical antiseptics remain underexplored. Most maxillofacial studies focus primarily on its antibacterial traits or long-term periodontal effects [11,12,13]. Although preliminary clinical studies have investigated various topical propolis preparations (such as mucosal gels, irrigants, or wound dressings) in oral wound healing, direct clinical evaluations comparing a standardized, alcohol-free propolis mouthwash to an active dual-agent regimen (CHX+BENZ) following third molar surgery remain scarce.

To address this gap, this prospective, randomized, double-blind clinical trial evaluated the therapeutic efficacy of a novel, non-alcoholic 3% propolis mouthwash on postoperative pain, edema, and trismus after impacted lower third molar extraction. To adhere to ethical standards, an active-control design (CHX+BENZ) was utilized instead of a placebo, ensuring all patients received standard prophylactic care to mitigate alveolar osteitis and infection risks.

## 2. Results

### 2.1. Demographic Data

**Figure 1:**
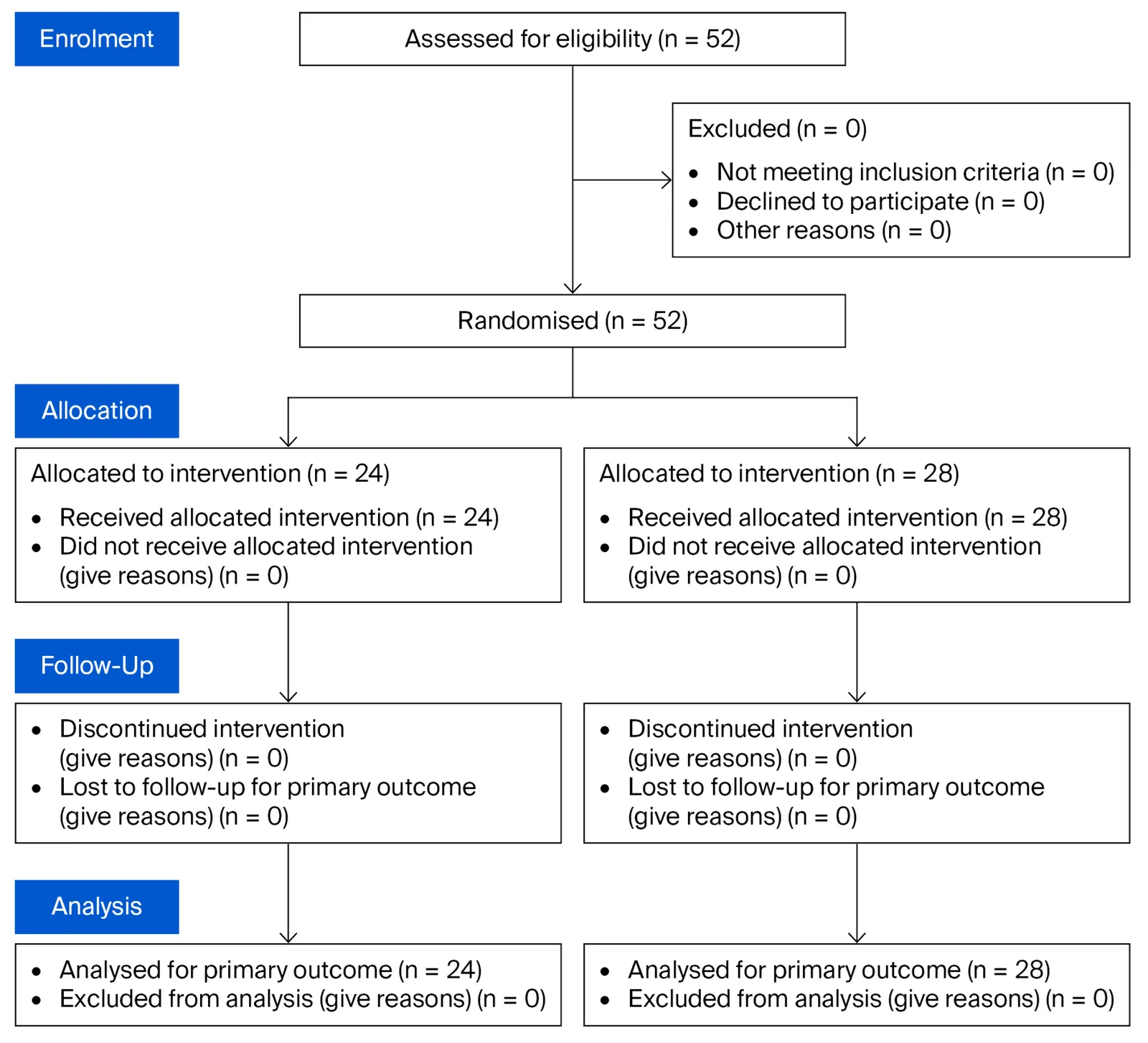
Flow diagram of the progress through the phases of a randomized trial of two groups (that is, enrolment, intervention allocation, follow-up, and data analysis).

The clinical trial safely concluded with a total cohort of 52 patients, comprising 38 females (73.1%) and 14 males (26.9%), with an overall mean age of 22.04 ± 4.05 years. Randomization successfully yielded homogenous groups: the CHX+BENZ group included 28 patients (20 females, 8 males; mean age: 22.25 ± 4.12 years), while the Propolis group consisted of 24 patients (18 females, 6 males; mean age: 21.80 ± 4.01 years). No statistically significant differences were observed between the two study arms regarding baseline age distribution or gender allocation (*p* > 0.05) (Table 1). The flow of participants through each stage of the trial is summarized in Figure 1.

### 2.2. Postoperative Pain (VAS Scores)

Longitudinal evaluations of subjective pain metrics revealed a predictable decline in intensity from postoperative days 2 to 7 across the entire study population (*p* < 0.001). In the intergroup analysis, no statistically significant differences in Visual Analog Scale (VAS) values were detected between the CHX+BENZ and Propolis groups on either the second (*p* = 0.723) or seventh (*p* = 0.804) day post-surgery. Subgroup analysis stratified by gender similarly showed no statistically significant differences in pain perception between the treatment modalities (*p* > 0.05) (Table 2). 

### 2.3. Trismus (Maximum Mouth Opening)

Mouth opening restriction parameters, measured as the percentage of baseline MMO, exhibited gradual physiological improvement over the follow-up timeline. On postoperative day 2, mean MMO percentages dropped to 64.41% ± 13.52% in the CHX +BENZ group and 64.21% ± 12.95% in the Propolis group, demonstrating a comparable early trismus effect (*p* = 0.954). By the seventh postoperative day, mouth opening capacity safely recovered to 86.17% ± 12.83% in the CHX+BENZ arm and 80.52% ± 10.94% in the Propolis arm. The intergroup differences regarding MMO recovery rates were not statistically significant on either days 2 or 7 (*p* > 0.05) (Table 3). 

### 2.4. Facial Edema Control

Facial swelling kinetics were calculated as percentage variations relative to individual baseline values. A significant therapeutic difference was identified on the second postoperative day. Patients utilizing the novel propolis mouthwash exhibited significantly lower facial edema accumulation (102.89% ± 1.31%) compared to those in the CHX+BENZ treatment arm (104.24% ± 1.82%; absolute difference: 1.35%, 95% CI: [0.47%, 2.23%]; Cohen’s d = 0.84; *p* = 0.004). This early anti-inflammatory attenuation favored propolis across the entire cohort. Exploratory sex-stratified analysis revealed a statistically significant difference in favor of propolis within the female subgroup (*p* = 0.032); however, given the small sample size in the partitioned male cohort (*n* = 14), these stratified observations should be interpreted with caution. By the seventh postoperative day, facial swelling resolved near baseline levels in both parallel arms, showing no statistically significant intergroup variances (*p* = 0.063) (Table 4). 

### 2.5. Clinical Postoperative Complications

Throughout the seven-day follow-up period, localized wound complications occurred in three patients (10.7%) in the CHX+BENZ arm (wound dehiscence with secondary alveolar osteitis) and in one patient (4.2%) in the Propolis arm (minor dehiscence with localized dry socket). Although fewer events were observed in the Propolis group, this difference was not statistically significant (Fisher’s exact test, *p* = 0.615; relative risk: 0.39; 95% CI: 0.04 to 3.49), representing an exploratory safety observation given the low overall event rate. Furthermore, analysis of total rescue analgesic consumption (paracetamol tablets) revealed no statistically significant difference between the CHX+BENZ group (6.61 ± 3.63 tablets; median: 6.00 [IQR: 4.00–10.00]) and the Propolis group (8.96 ± 4.52 tablets; median: 9.50 [IQR: 6.00–11.25]; Mann–Whitney U test, *p* = 0.056).

## 3. Discussion

Surgical removal of impacted wisdom teeth is a common outpatient maxillofacial procedure. Involving mucosal incision, flap elevation, and bone resection in the vascularized third molar area, this trauma triggers an inflammatory response resulting in edema, discomfort, and trismus [1,3], which markedly diminish patient quality of life [1,2]. Consequently, diverse therapeutic strategies are implemented to mitigate these sequelae [1,2,3,4,5]. Chlorhexidine gluconate mouthwashes—frequently combined with anti-inflammatory benzydamine hydrochloride—are standard post-extraction prescriptions. As a bisbiguanide formulation linked by a hexamethylene bridge, chlorhexidine is a potent antibacterial topical antiseptic used for decades [6,7,14]. Although studies prove its efficacy against plaque, gingivitis, oral lesions, and in promoting wound healing [14,15], its cytotoxicity and side effects—including taste alterations, desquamation, and tooth staining—necessitate safer alternatives.

Propolis, a resinous substance composed of plant resins, waxy materials, pollen, and essential oils gathered by honeybees [8,9], exhibits diverse biological activities, including antibacterial, antifungal, antiviral, anti-inflammatory, antiulcer, local anesthetic, antitumor, and immunostimulatory properties [16]. Consequently, it is widely utilized across medicine, dentistry, apitherapy, and biocosmetics.

This clinical study evaluated and compared the therapeutic efficacy of a propolis-containing mouthwash against a standard chlorhexidine gluconate and benzydamine hydrochloride combination (Kloroben) regarding postoperative morbidity following third molar surgery. Impacted mandibular third molar extraction induces a transient localized inflammatory response manifested as pain, trismus, and edema. Although chlorhexidine remains a conventional gold-standard antiseptic, its cytotoxicity and adverse effects underscore the clinical need for biocompatible natural alternatives.

To our knowledge, this is the first clinical study comparing the anti-inflammatory and wound-healing benefits of propolis and chlorhexidine gluconate mouthwashes following third molar surgery. The patented propolis formulation effectively alleviated postoperative pain and edema, while showing a numerical difference in surgical site healing and the frequencies of wound dehiscence and alveolitis. A key strength of this study design is using an active control (CHX+BENZ) rather than a placebo. Withholding proven antiseptic prophylaxis after third molar surgery presents ethical concerns due to infection and alveolar osteitis risks. By demonstrating that postoperative pain and trismus levels did not differ significantly between the groups, alongside superior early edema control in the Propolis group, this trial highlights the potential of propolis as a promising natural alternative in postoperative oral management.

Numerous studies have evaluated propolis in dentistry, focusing on caries, gingivitis, cavity sterilization, pulp inflammation, oral lesions, and the oral microbiota [16,17,18,19,20,21,22]. However, limited literature directly contrasts chlorhexidine and propolis, with comparative studies primarily focusing on their antibacterial properties [16,17,23,24,25]. Regarding these comparisons, Nazeri et al. [26] demonstrated the superior antibacterial efficacy of propolis over chlorhexidine in a rat model. Conversely, Dodway and Kukreja [27] found chlorhexidine more effective in inhibiting plaque formation in a 30-patient cohort. Additionally, Kuo et al. [28] reported that propolis mouthwash is safe and effective for managing cancer therapy-induced oral mucositis.

Waqar et al. [29] evaluated chlorhexidine and propolis mouthwashes alongside mechanical therapy in periodontitis patients over a 12-week follow-up, reporting that twice-daily use of both agents improved clinical parameters, with propolis showing greater efficacy in reducing bleeding on probing. Similarly, Anauate-Netto et al. [25] compared propolis, chlorhexidine, and placebo mouthwashes in 60 gingivitis patients, finding propolis superior in reducing gingival irritation and treating gingivitis. Extending to surgical wound healing, Moon et al. [30] evaluated topical propolis gel and gargle versus placebo post-tonsillectomy, demonstrating statistically significant improvements in pain control, hemorrhage reduction, and wound healing in the Propolis group.

In the present study, Visual Analog Scale (VAS) scores revealed no statistically significant differences in postoperative pain between CHX+BENZ gargle and Propolis gargle groups on postoperative days 2 and 7 (*p* > 0.05, Table 2), demonstrating that subjective pain alleviation followed a similar clinical pattern between the cohorts. The suppression of inflammatory cascades by propolis’s active constituents (such as CAPE) yielded postoperative pain and trismus values that did not significantly differ from those observed with the active control. While the benzydamine hydrochloride component in the CHX+BENZ formulation reduces pain by suppressing pro-inflammatory cytokines and prostaglandin synthesis, propolis exerts a regulatory effect on inflammatory pathways via its bioactive flavonoids and phenolics, such as CAPE and pinocembrin, which inhibit lipoxygenase and cyclooxygenase (COX-2) cascades resulting in no statistically significant differences in pain outcomes between the groups. Similarly, MMO percentage values for assessing trismus—triggered by pterygomasseteric muscle spasm—showed no significant intergroup differences on days 2 and 7 (*p* > 0.05, Table 3). Both cohorts demonstrated predictable chronological recovery, reaching approximately 80.52% to 86.17% of baseline mouth opening by day 7. This finding indicates that there were no statistically significant differences between the groups regarding the mitigation of inflammation within masticatory muscles and perimandibular tissues.

Considering chlorhexidine’s clinical limitations—including tooth staining, taste alterations, and mucosal desquamation—the lack of significant differences in clinical efficacy suggests that propolis warrants further investigation as a supportive option. Natural apitherapy agents offer favorable biocompatibility, minimizing oral fibroblast cytotoxicity while successfully managing postsurgical discomfort.

Beyond its wound-healing benefits, our formulated propolis gargle is exclusively water-based and alcohol-free, unlike many commercial mouthwashes. Furthermore, patients noted a mild local anesthetic effect in the surgical region following propolis gargle application. This inherent analgesic property reduces postoperative pain, suggesting that third molar surgery sequelae can be effectively managed with propolis mouthwash, as no significant differences in clinical recovery parameters were observed compared to conventional chemical agents.

Postoperative facial edema, typically peaking within 48 to 72 hours due to increased vascular permeability and fluid accumulation, was quantified using a reproducible three-line measurement technique. On postoperative day 2, the Propolis group demonstrated significantly less edema compared to the CHX+BENZ group (102.89 ± 1.31% vs. 104.24 ± 1.82% respectively; *p* = 0.004, Table 4), reflecting a large statistical effect size (Cohen’s d = 0.84). From a clinical standpoint, although an absolute difference of 1.35 percentage points represents a modest quantitative attenuation rather than a complete resolution of swelling, it indicates effective biological blunting of the acute inflammatory crest. This favorable early-phase anti-inflammatory activity can be attributed to multi-targeted cellular actions of active components like CAPE, which inhibit nuclear factor-kappa B (NF kappa B) activation alongside the arachidonic acid cascade. Downregulating inducible nitric oxide synthase (iNOS) and COX-2 expressions limits microvascular leakage more rapidly than conventional rinses. By day 7, edema resolved to near-baseline levels in both cohorts (100.33 ± 0.43% for Propolis and 100.68 ± 0.86% for CHX+BENZ; *p* = 0.063, Table 4), indicating synchronous late-stage healing.

Regarding postoperative rescue analgesic needs, total paracetamol intake did not differ significantly between the groups (median: 6.00 tablets in CHX+BENZ vs. 9.50 tablets in Propolis; *p* = 0.056), demonstrating that the propolis formulation provided a clinically acceptable level of comfort comparable to standard care without necessitating increased systemic analgesic support. Furthermore, while the incidence of localized wound complications (alveolar osteitis and wound dehiscence) was numerically lower in the Propolis group (4.2% vs. 10.7%), this difference did not achieve statistical significance (Fisher’s exact test, *p* = 0.615), representing an exploratory observation reflecting the low overall frequency of adverse events in this standardized cohort. Taken together, the novel non-alcoholic 3% propolis mouthwash presents a biocompatible therapeutic profile that effectively mitigates acute early-stage edema and supports physiological tissue healing following invasive oral surgery. 

A methodological consideration of this trial is that while the a priori sample size calculation was modeled on maximal mouth opening (MMO), the primary statistically significant intergroup divergence emerged in acute facial swelling on postoperative day 2. Although both parameters reflect localized inflammatory intensity, this positive anti-edematous finding should be confirmed in prospective clinical trials specifically powered for three-dimensional volumetric edema.

Despite these promising outcomes, certain limitations warrant acknowledgment. First, the modest sample size (*n* = 52), though statistically sufficient to detect early edema differences, limits generalizability, necessitating larger multi-center cohorts. Second, despite formulating the propolis mouthwash with peppermint essence and menthol to mask its characteristic flavor, and despite the parallel-group design precluding direct sensory cross-comparison by patients, the intrinsic herbal aroma of propolis represents a recognized inherent consideration in oral apitherapy trials. Nevertheless, because all primary and secondary anatomical measurements (digital caliper MMO and facial anatomical tape lines) were documented exclusively by an independent, fully blinded clinician, observer bias was systematically prevented. Third, comprehensive quantitative chromatographic fingerprinting analysis (e.g., HPLC-DAD or LC-MS/MS) of the propolis extract—specifically regarding total phenolic and flavonoid contents and specific active components such as CAPE—was not performed; this constitutes a limitation that should be addressed in future studies. Fourth, although sex-stratified comparisons indicated pronounced edema reduction in female patients, these subgroup evaluations were conducted on small partitioned cohorts, limiting statistical power. Furthermore, analyzing clinical parameters across individual post-surgical time points carries an inherent risk of Type I error inflation. Therefore, both sex-specific nuances and individual day-2 differences must be viewed as exploratory findings that require validation in larger, prospectively stratified trials utilizing longitudinal and interaction modeling. Finally, as a natural apitherapy product, propolis exhibits minor geographic and seasonal chemical variations. Although standardized extracts were utilized, future multi-centric trials with extended follow-ups are needed to optimize concentration and validate molecular mechanisms in wound healing.

Future investigations should incorporate larger, multi-center cohorts to improve statistical sensitivity when evaluating rare adverse outcomes such as dry socket and wound dehiscence. In addition, prospective clinical protocols combining objective macroscopic measurements with quantitative biomarker analyses—such as tracking salivary or gingival crevicular fluid levels of pro-inflammatory cytokines (e.g., IL-1*β*, IL-6, TNF-*α*, and PGE2)—are warranted to elucidate the precise molecular mechanisms driving the early anti-edematous properties of propolis. Exploring varying concentrations and application regimens could also help optimize formulation kinetics and therapeutic outcomes in oral surgical settings.

## 4. Materials and Methods

### 4.1. Study Design and Ethical Approval

This study was designed as a prospective, randomized, active-controlled, double-blind clinical trial. The study protocol was certified by the Ethics Committee of Ataturk University (Ethical Number 2020/3-4, approved on 26 March 2020). All participants volunteered to participate in the study after giving their informed consent. Patient recruitment was carried out between November 2020 and October 2021. All prospective participants received detailed oral and written information regarding the surgical procedures and follow-up protocols, and signed voluntary informed consent forms prior to enrollment. All primary and secondary clinical endpoints were predefined in the original ethical submission prior to patient enrollment. To safeguard intellectual property during ongoing national patent evaluation of the novel propolis formulation, and in accordance with updated international clinical trial reporting guidelines, the trial was registered retrospectively with ClinicalTrials.gov (Identifier NCT07364253 registered 16 January 2026) without altering prespecified endpoints or investigative procedures. This clinical investigation was financially supported by the Scientific Research Projects (BAP) coordination unit of Atatürk University (Project Funding Number: 2020/8652). This study was conducted and reported in accordance with the CONSORT guidelines (Figure 1).

### 4.2. Patient Selection and Eligibility Criteria

A total of 52 healthy outpatients who required surgical extraction of a single impacted mandibular third molar were enrolled from the Department of Oral and Maxillofacial Surgery at Atatürk University, Faculty of Dentistry. To ensure sample homogeneity, all target third molars were clinically and radiographically classified as Class II, Position B, with a mesioangular angulation based on the Pell and Gregory and Winter classification systems. 

All selected participants were categorized under the American Society of Anesthesiologists (ASA) Physical Status Classification System as ASA I, indicating healthy individuals without systemic diseases. The exclusion criteria were defined as follows: patients presenting with acute local infection, a history of acute pericoronitis, or fascial space abscess at the surgical site; patients with known hypersensitivity to bee products, propolis, or chlorhexidine; heavy smokers; and pregnant or lactating women. 

### 4.3. Sample Size Calculation and Randomization

The primary outcome measure in this trial was trismus (evaluated as the percentage of baseline maximal mouth opening, MMO), while facial edema and subjective pain (VAS scores) served as secondary outcomes. The a priori sample size calculation was conducted using G*Power software (v3.1.9.4) based on MMO values reported by Gümrükçü [1]. Assuming a large effect size (d = 0.734), a two-tailed significance level (∝) of 0.05, and a standard statistical power (1 − *β*) of 0.80 (80%), the calculation indicated a minimum sample size of 48 patients (24 per group). To compensate for potential dropouts, a total of 52 patients were successfully enrolled and randomized into two parallel treatment arms: the 3% Propolis group (*n* = 24) and the CHX+BENZ group (*n* = 28). 

Randomization was executed using a computer-generated random allocation sequence, and allocation concealment was maintained using sequentially numbered, opaque, sealed envelopes. The primary operating surgeon detailed the postoperative instructions but was completely blinded to the randomized group assignments during data entry. All clinical preoperative and postoperative measurements were meticulously recorded by an independent assistant physician who remained entirely blinded to the patient allocation arms throughout the follow-up period.

### 4.4. Formulation and Characterization of the 3% Propolis Mouthwash

**Figure 2:**
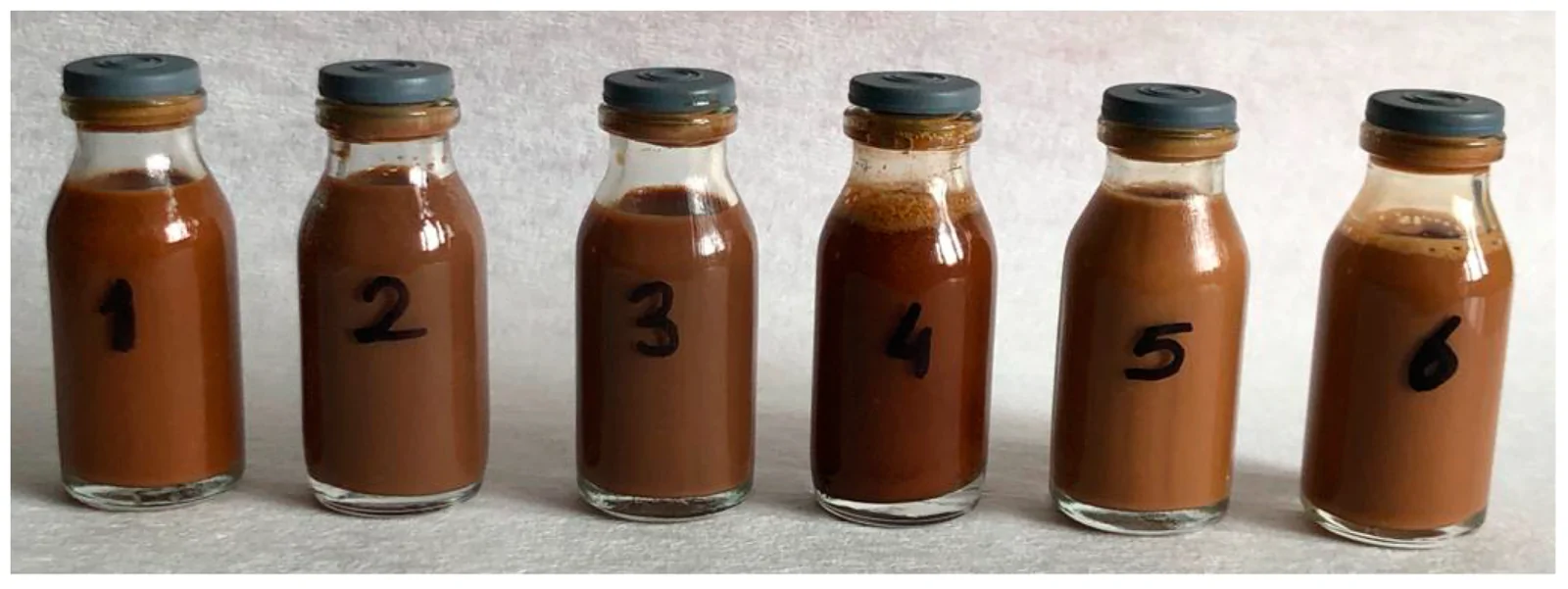
Preformulation experiments (Figure 2 is completely original and was created entirely by the authors).

The novel propolis-containing mouthwash was custom-developed through collaborative preformulation experiments conducted in the Departments of Pharmacology (Faculty of Medicine and Pharmaceutical Technology, Faculty of Pharmacy) at Atatürk University (Figure 2). Raw propolis was harvested from *Apis mellifera* apiaries located in the Eastern Anatolia region (Erzurum, Turkey). Solid raw propolis was extracted utilizing 96% ethanol(Sigma-Aldrich, St. Louis, MO, USA) at a 1:10 (*w*/*v*) ratio under magnetic stirring (750–1000 rpm) for 30 minutes, yielding an ethanolic propolis extract with an extraction yield of approximately 68.5% *w*/*w*. 

To overcome the inherent water-insolubility of raw propolis, an amount of solid extract corresponding to 4.5 g of the initial crude propolis was initially extracted utilizing 96% ethanol to yield a standardized extract. This extract was dissolved in specific solvent ratios using a heated magnetic stirrer (RH basic 2, IKA, Staufen, Germany) operating at 750–1000 rpm for durations of 5, 10, 15, and 30 minutes. Crucially, during the final formulation refinement, the volatile ethanol solvent was completely evaporated (for 10 min, 60 rpm, and 40–45 °C) to ensure a non-alcoholic end product. 

The formulation was then stabilized by incorporating viscosity-enhancing agents [propylene glycol (Sigma-Aldrich, St. Louis, MO, USA) and 70% D-sorbitol (Sigma-Aldrich, Saint-Quentin-Fallavier, France)] into an aqueous carrier. Aromatic compounds [peppermint essence (Doğa İlaç, Istanbul, Turkey) and menthol (Merck, Darmstadt, Germany)] were solubilized using a mild surfactant [Tween 20 (ISOLAB, Eschau, Germany)] and integrated to optimize organoleptic acceptability, and sweeteners [sucralose (Sigma-Aldrich, Taufkirchen, Germany)] were added to enhance taste. To maintain microbial stability, a preservative [sodium benzoate (Acros Organics, Mumbai, India)] was added, and the pH was adjusted to human salivary pH (6.5–7.0) using citric acid (Fluka, Vienna, Austria). The final mouthwash volume was adjusted to 150 mL utilizing distilled water (Merck Millipore, Darmstadt, Germany) (Table 5).

Characterization controls confirmed excellent compatibility with intraoral physiology. To verify chemical stability, absorbance profiles of the propolis solutions were scanned between 200 and 500 nm using a UV-VIS spectrophotometer (Beckman Coulter, Brea, CA, USA) showing identical maximal peak wavelengths across batches, indicating that no molecular alterations were induced by the excipients. 

To ensure rigorous double-blinding, both the newly formulated propolis mouthwash [enriched with mint essence (Doğa İlaç, Istanbul, Turkey) and menthol (Merck, Darmstadt, Germany) for organoleptic masking] and the active control CHX+BENZ solution were transferred into identical, unlabeled, opaque plastic bottles by an independent researcher who was not involved in patient allocation, surgical procedures, or follow-up evaluations. The bottles were coded with random identifiers (e.g., Mouthwash A and Mouthwash B), and the allocation key was sealed in an opaque envelope until all statistical analyses were finalized. Given the parallel-group trial design, each patient received only their assigned coded bottle without access to or comparative familiarity with the alternative formulation. Consequently, the operating surgeon, the participants, and the independent assistant clinician responsible for all clinical measurements (MMO and facial measurements) remained strictly blinded to the group assignments throughout the study.

### 4.5. Surgical Protocol and Postoperative Care

All surgical extractions were performed by the same experienced oral surgeon to eliminate operator variability. All operations were performed under local anesthesia following a standardized surgical protocol, with anatomical difficulty classified according to the Pederson index [31]. Local anesthesia was induced via standard inferior alveolar, lingual, and buccal nerve blocks using 4 mL of articaine hydrochloride with 1:100,000 epinephrine (Ultracaine D-S Fort, Sanofi Aventis, İstanbul, Turkey). A full-thickness triangular mucoperiosteal flap was elevated to expose the impacted tooth. Bone removal (osteotomy) and tooth sectioning (odontosection) were executed under continuous sterile saline irrigation using a rotary handpiece (W&H Dentalwerk, Bürmoos, Austria) and surgical burs (Meisinger, Neuss, Germany). Following tooth removal, meticulous curettage of the socket was performed to eradicate any residual dental follicle tissues. Primary wound closure was achieved using 3-0 silk sutures (Boz, Ankara, Turkey). 

Postoperatively, standard systemic pharmacotherapy was prescribed uniformly to all patients: oral amoxicillin combined with clavulanic acid (Augmentin, GlaxoSmithKline, Brentford, UK, 2 g daily for 5 days) and oral paracetamol (Parol, Atabay, Istanbul, Turkey, 1 g, up to 4 times daily as rescue analgesic for 3 days). Active mouthwash therapies commenced on the second postoperative day to prevent initial clot displacement. 

Patients in the study arm used the custom-formulated non-alcoholic 3% propolis mouthwash twice daily for 1 minute, while patients in the control arm were instructed to rinse with a commercially available combination mouthwash containing 0.12% chlorhexidine gluconate and 0.15% benzydamine hydrochloride (Kloroben^®^, Drogsan Pharmaceuticals, Ankara, Turkey) twice daily for 1 minute.

Both mouthwash regimens were maintained until the seventh postoperative day, at which point all remaining sutures were surgically removed. 

### 4.6. Clinical Assessment Parameters

Clinical parameters were meticulously recorded at three distinct time points: preoperatively (baseline), and on postoperative days 2 and 7.

- Postoperative Pain: Subjective pain intensity was quantified utilizing a standard 10 cm Visual Analog Scale (VAS), where 0 indicated "no pain" and 10 represented "unbearable pain". The total number of rescue analgesic tablets (Parol)consumed by each patient was also documented.
- Facial Edema: Facial swelling was evaluated using a flexible paper tape ruler (Seca GmbH & Co. KG, Hamburg, Germany) to measure the following facial anatomical reference lines: (1) tragus to the labial commissure, (2) tragus to the soft tissue pogonion, and (3) outer canthus of the eye to the mandibular angle [32]. Measurements were taken twice by a single calibrated examiner blinded to group assignment and averaged. The cumulative sum of these three distances was calculated at baseline (S_0_) and postoperatively on days 2 and 7 (S_t_). Postoperative edema was determined using the following formula:
  (1) $$\mathrm{Edema}\left(\%\right)=\frac{\mathrm{St}}{S0}\times 100$$
  Intra-observer reproducibility assessed on repeated baseline measurements demonstrated high reliability (intraclass correlation coefficient, ICC = 0.94).
- Trismus: MMO was determined by measuring the maximum interincisal distance between the upper and lower central incisors at maximum opening using a digital caliper (Insize, Suzhou, China) [33]. Trismus is expressed as a percentage of limitation compared to baseline MMO.
- Complications: Flap health, wound dehiscence, presence of alveolar osteitis (dry socket), and soft-tissue healing characteristics were evaluated and recorded on postoperative days 2 and 7.

### 4.7. Statistical Analysis

Statistical analysis was performed using SPSS (v23.0; IBM Corp., Armonk, NY, USA), and data normality was assessed via Shapiro–Wilk and Kolmogorov–Smirnov tests. Baseline demographics were compared using Chi-Square or independent samples *t*-tests. Since clinical parameters were non-normally distributed, non-parametric tests were applied: Mann–Whitney U tests for intergroup comparisons (VAS, MMO, edema) at each time point, and Friedman tests with post hoc Wilcoxon signed-rank tests for intragroup longitudinal variations. Statistical significance was set at *p* < 0.05. Continuous variables are presented as mean ± standard deviation (SD) alongside median and interquartile range (IQR, 25th–75th percentiles) to reflect data distribution accurately. Categorical variables, including complication rates (alveolar osteitis and wound dehiscence), were evaluated using Fisher’s exact test. Total rescue analgesic consumption was compared between groups using the Mann–Whitney U test. Exploratory subgroup comparisons across individual postoperative follow-up intervals were interpreted without family-wise adjustments, accounting for the potential risk of Type I error inflation.

## 5. Conclusions

Within the limitations of this clinical trial involving young, healthy ASA-I patients, an alcohol-free 3% propolis oral rinse demonstrated no statistically significant differences in short-term outcomes when compared to standard chlorhexidine gluconate and benzydamine hydrochloride combinations following mandibular third molar surgery. No statistically significant differences were detected between the Propolis and CHX+BENZ groups regarding postoperative pain intensity, maximal mouth opening recovery, or rescue analgesic requirements. Most notably, propolis was associated with a statistically smaller facial edema measurement on the 2nd postoperative day, alongside a favorable localized tissue healing profile. Given the modest absolute difference in acute swelling and the single-center design, larger, prospectively registered clinical trials with extended follow-up are required to substantiate these preliminary findings before routine adoption in maxillofacial surgical practice can be advocated.

## Figures and Tables

**Table 1:** Demographic characteristics of the study participants.

Parameter	Total Cohort (*n* = 52)	CHX+BENZ Group (*n* = 28)	Propolis Group (*n* = 24)	*p*-Value
Age (years)				
Mean ± SD	22.04 ± 4.05	22.25 ± 4.12	21.80 ± 4.01	>0.05
Gender, *n* (%)				>0.05
Female	38 (73.1%)	20 (71.4%)	18 (75.0%)	
Male	14 (26.9%)	8 (28.6%)	6 (25.0%)	

*p*: significance, SD: standard deviation.

**Table 2:** Intergroup evaluation in terms of VAS values.

		VAS-Day 2			VAS-Day 7		
	Age (Mean ± SD)	CHX+BENZ Gargle (28)	Propolis Gargle (24)	*p*	CHX+BENZ Gargle (28)	Propolis Gargle (24)	*p*
Male (14)	20.93 ± 1.94	2.03 ± 0.97 (2.10 [1.12–2.92])	2.64 ± 1.67 (2.35 [1.80–4.05])	0.446	0.17 ± 0.32 (0.00 [0.00–0.15])	0.09 ± 0.16 (0.00 [0.00–0.07])	0.556
Female (38)	22.45 ± 4.55	3.75 ± 1.77 (3.15 [2.60–5.00])	3.49 ± 1.88 (3.35 [2.40–4.50])	0.663	0.66 ± 0.68 (0.35 [0.20–1.00])	0.90 ± 1.61 (0.00 [0.00–0.93])	0.588
All patients (52)	22.04 ± 4.05	3.38 ± 1.77 (3.00 [2.38–4.55])	3.20 ± 1.82 (3.25 [2.22–4.33])	0.723	0.56 ± 0.65 (0.30 [0.18–0.85])	0.63 ± 1.36 (0.00 [0.00–0.33])	0.804

SD: standard deviation, VAS: Visual Analog Scale. Data are presented as mean ± SD (median [25th–75th interquartile range]). *p*: Mann–Whitney U test.

**Table 3:** Intergroup evaluation in terms of maximum mouth opening (%MMO).

		% MMO-Day 2			% MMO-Day 7		
	Age (Mean ± SD)	CHX+BENZ Gargle (28)	Propolis Gargle (24)	*p*	CHX+BENZ Gargle (28)	Propolis Gargle (24)	*p*
Male (14)	20.93 ± 1.94	71.59 ± 10.94 (69.88 [69.77–79.73])	66.61 ± 12.40 (65.33 [60.00–70.16])	0.450	93.22 ± 8.79 (97.50 [85.95–100.00])	84.37 ± 11.18 (86.46 [76.31–91.52])	0.136
Female (38)	22.45 ± 4.55	62.46 ± 13.70 (59.97 [51.47–72.64])	63.00 ± 13.45 (61.44 [51.11–70.21])	0.904	84.24 ± 13.24 (84.06 [75.44–96.67])	78.60 ± 10.65 (78.57 [71.31–86.05])	0.169
All patients (52)	22.04 ± 4.05	64.41 ± 13.52 (66.25 [52.57–73.30])	64.21 ± 12.95 (63.11 [53.00–70.16])	0.954	86.17 ± 12.83 (87.51 [78.11–98.26])	80.52 ± 10.94 (80.54 [72.23–90.23])	0.097

MMO: maximal mouth opening, CHX: chlorhexidine glukonat, BENZ: benzydamine hydrochloride, SD: standard deviation. Data are presented as mean ± SD (median [25th–75th interquartile range]). *p*: Mann–Whitney U test.

**Table 4:** Intergroup evaluation in terms of edema (% change).

		% Edema-Day 2			% Edema-Day 7		
	Age (Mean ± SD)	CHX+BENZ Gargle (28)	Propolis Gargle (24)	*p*	CHX+BENZ Gargle (28)	Propolis Gargle (24)	*p*
Male (14)	20.93 ± 1.94	103.72 ± 1.87 (103.03 [102.63–105.34])	102.36 ± 0.85 (102.32 [101.83–102.73])	0.146	100.54 ± 0.55 (100.53 [100.07–100.81])	100.29 ± 0.34 (100.26 [100.00–100.33])	0.305
Female (38)	22.45 ± 4.55	104.39 ± 1.83 (103.98 [103.25–105.42])	103.15 ± 1.44 (103.23 [102.17–103.90])	0.032 *	100.72 ± 0.94 (100.28 [100.00–101.33])	100.35 ± 0.48 (100.26 [100.00–100.54])	0.120
All patients (52)	22.04 ± 4.05	104.24 ± 1.82 (103.80 [102.82–105.73])	102.89 ± 1.31 (102.48 [101.93–103.51])	0.004 **	100.68 ± 0.86 (100.28 [100.00–101.15])	100.33 ± 0.43 (100.26 [100.00–100.52])	0.063

SD: standard deviation, CHX: chlorhexidine gluconate, BENZ: benzydamine hydrochloride. Data are presented as mean ± SD (median [25th–75th interquartile range]). *p*: Mann–Whitney U test. * *p* < 0.05, ** *p* < 0.01.

**Table 5:** Ingredients and quantities used in the formulation.

Ingredients and Quantities Used in the Formulation (*w*/*v*)	
Propolis	3% (4.5 g/150 mL)
Propylene glycol	45 mL
D-sorbitol (70%)	5 mL
Sucralose	0.3 g
Sodium benzoate	0.15 g
Tween 20	0.5 mL
Peppermint essence	5 mL
Menthol	0.15 g
Citric acid	0.15 g
Distilled water	94.5 mL
Total = 150 mL	

## Data Availability

The original contributions presented in this study are included in the article. Further inquiries can be directed to the corresponding author.

## Abbreviations

VAS	Visual analog scale
CAPE	Caffeic acid phenethyl ester
LOX	Lipoxygenase
COX	Cyclooxygenase
ASA	American society of anesthesiologists
MMO	Maximum mouth opening
